# Gadd45 in stress signaling

**DOI:** 10.1186/1750-2187-3-15

**Published:** 2008-09-12

**Authors:** Dan A Liebermann, Barbara Hoffman

**Affiliations:** 1 Fels Institute for Cancer Research & Molecular Biology, & Department of Biochemistry, Temple University School of Medicine, Philadelphia, PA 19140, USA

## Abstract

Gadd45 genes have been implicated in stress signaling in response to physiological or environmental stressors, which results in cell cycle arrest, DNA repair, cell survival and senescence, or apoptosis. Evidence accumulated implies that Gadd45 proteins function as stress sensors is mediated by a complex interplay of physical interactions with other cellular proteins that are implicated in cell cycle regulation and the response of cells to stress. These include PCNA, p21, cdc2/cyclinB1, and the p38 and JNK stress response kinases. What deterministic factors dictate whether Gadd45 and partner proteins function in either cell survival or apoptosis remains to be determined. An attractive working model to consider is that the extent of cellular/DNA damage, in a given cell type, dictates the association of different Gadd45 proteins with particular partner proteins, which determines the outcome.

## Background

### The cellular response to stress

The cellular response to environmental and physiological stress is very complex, encompassing a myriad of molecular pathways, with a plethora of regulators and effectors. Both environment agents [1] and physiological processes involving activated oxygen species and other reactive agents [2] can damage DNA. Genotoxic stress, resulting in DNA damage, is an inescapable aspect of life with which mammalian cells have to contend. Two common environmental agents that cause cellular injury primarily by damaging DNA are ionizing radiation, such as x-rays, and nonionizing UV radiation [1]. X-rays damage DNA primarily by generating hydroxyl radicals, also produced by oxidative stress, which alter the structure of bases, and the resulting DNA damage is repaired by various base-excision repair mechanisms [1]. Lesions caused by UV radiation are primarily cyclobutane pyrimidine dimers & 6-4 photoproducts that are repaired by nucleotide-excision repair [1]. Alkylating agents, such as methyl methanesulfonate (MMS), represent a major class of chemical agents that damage DNA [3]. Damage caused by alkylating agents is repaired by either base-excision repair mechanisms as well, or by in-situ repair [1].

Aberrations in DNA repair pathways are the major molecular pathology in several cancer prone hereditary diseases [4]. Defects in DNA repair pathways are known to be an early step in oncogenesis, accelerating its progression [4-7]. Paradoxically, DNA-damage inducing agents that can cause tumors, such as x-irradiation and a variety of alkylating agents, are frequently used in cancer therapy [8-11]. In recent years it became evident that both the molecular basis for the initial susceptibility of cancer cells to anti-cancer genotoxic drugs, and the development of treatment resistance originate from genetic lesions that alter the function of genes playing roles in negative growth control, notably in determining cell cycle arrest and apoptotic set points [9]. Thus, understanding the molecular-genetic pathways that mediate the response of mammalian cells to genotoxic stress as well as other types of stress, is of high priority from both basic science and clinical points of view.

Mammalian cells have evolved an elaborate defense mechanism to maintain genomic integrity by preventing the fixation of permanent damage from genotoxic stress. This includes activation of cell cycle arrest checkpoints at the G1/S and G2/M transitions [12-14] and activation of a cell death program [9,10]. Whether a particular cell undergoes either cell cycle arrest, to allow damaged DNA to be repaired [15], or rapid apoptosis depends on the extent of genotoxic damage and the cell type. For example, hematopoietic cells readily undergo apoptosis following exposure to doses of g-irradiation that induce cell cycle arrest in fibroblasts [9,10,16]. The ATM/p53 tumor suppressor pathway [17-19] and the p38/JNK kinase pathways [20-23] have recently been determined to play major roles as mediators of the mammalian response to genotoxic stress. However, the molecular/genetic circuitries of stress response pathways and their mode of action have not yet been fully delineated. For example, how such pathways interact to signal either cell cycle arrest or programmed cell death is still not clearly understood. This review is targeted at assessing the role the Growth Arrest DNA Damage (Gadd) 45 genes play as sensors of cellular stress.

### Mode of action of Gadd45 proteins: Coordination of cellular stress responses by interaction with partner proteins

**Figure 1:**
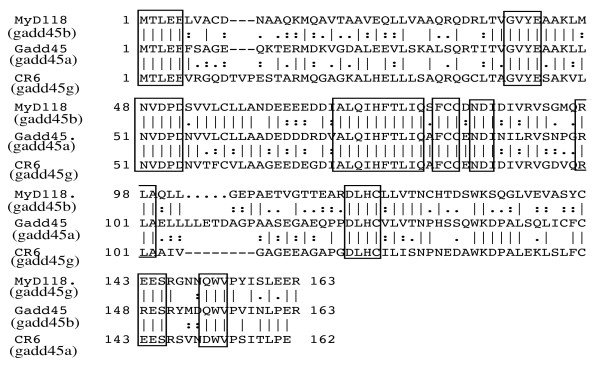
**The Gadd45 family of proteins**. Alignment of the amino acid sequences of murine MyD118 (Gadd45b), murine Gadd45 (Gadd45a) and murine CR6 (gadd45g). Conserved amino acid regions are boxed.

Gadd45 genes were cloned in this laboratory [24,25], in the laboratory of Dr. Smith [26] and in the Fornace laboratoy at the NIH [27]. Gadd45, MyD118, and CR6 (currently termed Gadd45a, Gadd45b, & Gadd45g, respectively, and collectively referred to as Gadd45 proteins, gadd45 genes or Gadd45 family) encode for small (18 kd), evolutionarily conserved proteins that are similar to each other (55%–57% overall identity at the amino acid level), are highly acidic (pI = 4.0–4.2), and are localized primarily within the cell nucleus [24-38] (Fig. 1).

**Figure 2:**
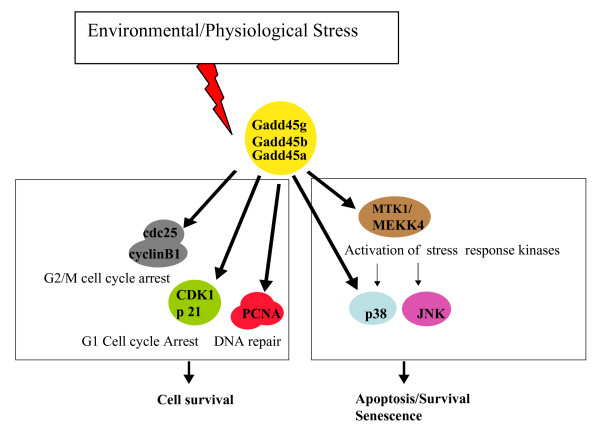
**Gadd45 in stress signaling**. Coordination of cellular stress responses via interaction with partner proteins.

Evidence accumulated in recent years implies that Gadd45 proteins function as stress sensors, is mediated by a complex interplay of physical interactions with other cellular proteins that are implicated in cell cycle regulation and the response of cells to stress (Fig. 2).

#### PCNA

It was shown that all three Gadd45 proteins interact with PCNA [39-43], a nuclear protein that plays a central role in DNA repair and replication [[44-46] & therein]. Direct evidence was obtained that interaction of either gadd45a or gadd45b with PCNA participates in nucleotide excision DNA repair [39-41]. This has not yet been yet established for gadd45g.

#### p21

All three Gadd45 proteins were observed to interact with p21 [39,43], a p53 target gene that is a universal cyclin dependent kinase inhibitor implicated in G1/S and G2/M cell cycle arrest [44-49]. The role for this interaction in cell cycle control remains to be elucidated.

#### Cdc2/cyclinB1

In normal cells p21 and PCNA are found in quaternary complexes that also include cyclins and cyclin dependent kinases (cdks) [47-52]. It was, therefore, logical to test whether any one of the Gadd45 family proteins interact with Cdk/cyclin complexes. It was observed that all three Gadd45 proteins specifically interact with the cdc2 (cdk1)/cyclinB1 complex [38,53-55]. Association of either Gadd45a or Gadd45b proteins with cdc2/cyclinB1 results in dissociation of the cdc2/cyclinB1 complex that, in turn, inhibits cdc2 kinase activity [38]. In contrast, gadd45g inhibits cdc2/cyclinB1 kinase activity without disrupting the complex [38]. Since cdc2/cyclinB1 regulates the transition of cells from G2 to the M phase of the cell cycle [56,57], these findings implicate the interaction of Gadd45 proteins with cdc2 in the activation of the G2/M checkpoint in response to certain genotoxic stresses [38,58]. Using RKO lung carcinoma cell lines that express antisense gadd45 RNAs, it was shown that all three Gadd45 proteins cooperate in activation of S and G2/M checkpoints following exposure of cells to UV irradiation [38]. Interestingly, myeloid enriched BM cells deficient for either gadd45a or gadd45b were defective in G2/M arrest following exposure to UVC and VP-16, but not to daunorubicin. This indicates the existence of different G2/M checkpoints, either dependent or independent of gadd45. The molecular basis for this phenomena remains to be elucidated. Also, the role of Gadd45g in modulating G2/M checkpoints in response to different genotoxic stress agents needs to be investigated.

### MAPK stress kinases

Interaction of Gadd45 proteins with MEKK4, an upstream activator of the stress induced p38/JNK kinases, and the resulting stimulation of its kinase activity, has been implicated in stress induced p38 and JNK activation [33]. Evidence was obtained that Gadd45b & Gadd45g activate p38/JNK signaling and cytokine production in effector T cells [59,60]. That Gadd45 proteins can directly interact with and activate p38 kinase was observed as well [[61] & unpublished]. It is possible that interaction of Gadd45 proteins with p38 and its ensuing activation plays an ancillary function to interaction with the upstream regulator MEKK4. On the other hand, TNFa-NF-kB mediated induction of Gadd45b and its association with MKK7 has been shown to blunt JNK activation [62]. UV induced Gadd45b has also been implicated in blunting JNK activation by modulating MKK4 activity [63]. These observations, made in different cell types responding to different stress stimuli, suggest that Gadd45 mediated modulation of stress response MAPK activity is both cell type and stimulus specific.

### Gadd45 in cellular stress responses

Gadd45 family members are rapidly induced by genotoxic stress agents [38,64,65], as well as by terminal differentiation and apoptotic cytokines [24,25,30,32]. Emerging evidence indicates that the proteins encoded by these genes play pivotal roles as stress sensors that modulate and integrate the response of mammalian cells to a variety of environmental and physiological stressors [25,27-29], either dependent or independent of p53 [34,35,66]. They also function to modulate tumor formation in response to oncogenic stress [67]. Gadd45 proteins appear to serve similar, but not identical, functions along different stress response pathways. For example, only gadd45b is induced by TGF-b [68,69], whereas only Gadd45a is a p53 target [70-72]. All three genes are induced with different expression kinetics during terminal hematopoietic differentiation, associated with growth arrest and apoptosis [25]. Distinct expression patterns for these genes were also observed in a variety of murine tissues [25,32]. Importantly, individual members of the Gadd45 family are differentially induced by a variety of genotoxic and environmental stress agents [25,32,33,73-75], indicating that each gene is induced by a distinct subset of environmental stresses. To what extent the function of each of the Gadd45 proteins is unique or overlaps with the functions of the other proteins, remains to be determined.

As detailed below, gadd45 genes have been implicated in the control of cell cycle arrest[25,26,28,38,58], DNA repair[39-41], cell survival[41,63,76-79], apoptosis[33,68,75,69,42,43,80-82], senescence[67], and susceptibility of cells for transformation in vitro and in tumor development in vivo[67,83].

#### Cell cycle arrest

Inhibiting endogenous expression of gadd45a, gadd45b, or gadd45g in human cells by antisense gadd45 constructs was found to impair the G2/M checkpoint following exposure to UV radiation or MMS [28,38,58], and microinjecting a gadd45a expression vector into primary human fibroblasts arrested the cells at the G2/M boundary of the cell cycle [58]. In another study [31] deregulated ectopic expression of CR6 (gadd45g) in HeLa cells had little effect on HeLa cell growth under normal culture conditions. However, following serum withdrawal gadd45g blocked HeLa cell G2/M transition and caused endoreduplication. In contrast, under normal culture conditions in USO2 cells, ectopic expression of any one of the Gadd45 proteins resulted in blocking either G1/S or G2/M transitions [31]. In this laboratory it was observed that IPTG-induced ectopic expression of gadd45a, gadd45b, or gadd45g in both H1299 and M1 cells, in the absence of genotoxic stress, retarded cell growth and increased accumulation of cells in the G1 phase of the cell cycle [80]. G2/M cell cycle arrest mediated by Gadd45 was shown to be due to their ability to interact with and inhibit the kinase activity of the cdc2/cyclinB1 complex [38,53,58]. Their ability to arrest cells in G1 is less well understood. It is possible that interaction of Gadd45 proteins with p21 plays a role in G1 cell cycle arrest; however, it has not been established if this is the case.

#### DNA repair

Evidence was obtained that Gadd45a and Gadd45b function in DNA excision repair through their interactions with PCNA [39-41]. Further assessment of the role Gadd45a plays in DNA repair pathways showed that it functions in global genomic repair (GGR) [41], which is a sub-pathway of nucleotide excision repair (NER). Interestingly, recent evidence suggests a role for Gadd45a in the promotion of epigenetic gene activation by repair-mediated DNA demethylation [84]. Whether Gadd45g plays a role in DNA repair has not been established.

#### Apoptosis

Ample evidence exists that Gadd45 proteins have a pro-apoptotic function. For example, it was observed that blocking MyD118 (Gadd45b) by antisense expression in M1 myeloblastic leukemia cells impaired TGFb-induced cell death, thereby implicating Gadd45b as a positive modulator of TGFb-induced apoptosis [68,69]. Consistent with this notion, IPTG-inducible ectopic expression of Gadd45b accelerated TGFb-induced apoptosis in M1 cells [32]. More recently, it was demonstrated that TGFb-induced apoptosis is mediated by Gadd45b via p38 activation in primary hepatocytes from wild type mice, and was blocked in hepatocytes from Gadd45b-/- mice [69]. Furthermore, ectopic expression of all three Gadd45 proteins was shown to induce apoptosis in HeLa cells [33], and to enhance stress mediated apoptosis in both M1 leukemia and H1299 lung carcinoma cells [42,43,75]. Also, BRCA-1-mediated induction of gadd45a has been implicated in apoptosis of breast cancer cells [85], whereas gadd45g expression was shown to have a role in neuronal cell death [81]. Furthermore, gadd45a has also been implicated in apoptosis of UV-irradiated keratinocytes [82].

#### Survival

Intriguingly, in apparent contradiction to the role Gadd45 proteins play in cell death, many observations are consistent with a role in cell survival. Clonogenic survival assays with gadd45a-/- MEFs [41] and RKO cells expressing antisense gadd45a RNA [76] showed that deficiency in gadd45a increases the sensitivity of cells to killing by UV irradiation or cisplatin. It has been suggested that Gadd45b plays a role in TNFa-NFkb mediated cell survival of mouse embryo fibroblasts [77], although other data have challenged this view [78]. In addition, it was recently documented that gadd45a and gadd45b deficiency each sensitized hematopoietic cells to genotoxic stress induced apoptosis [79]. It was shown that in hematopoietic cells exposed to UV radiation Gaddd45a and Gadd45b cooperate to promote cell survival by two distinct signaling pathways involving activation of a novel gadd45a mediated p38-NF-kB-mediated survival pathway and Gadd4545b-mediated inhibition of the stress response MKK4-JNK pathway [63]. The role Gadd45 proteins play in DNA repair and cell cycle arrest is compatible with a survival function. Consistent with the idea that interaction of Gadd45 proteins with PCNA may promote cell survival by enhancing DNA repair, it was observed that Gadd45/PCNA interaction impedes their apoptotic function [42,43].

#### Senescence

Senescence represents a physiological stress response associated with cellular aging (90). When primary mammalian cells are cultured in vitro they undergo a limited number of cell divisions and then arrest in a state known as replicative senescence. Such cells are irreversibly arrested in the G1 phase of the cell cycle and are no longer sensitive to growth factor stimulation [[85,86] & therein]. Senescence is a barrier that cells must overcome in order to become immortal and proliferate indefinitely, and, therefore, functions as a tumor-suppressing mechanism that limits the proliferative capacity of cells in vivo [85,86]. Expression of various oncogenes in cultured primary cells induces a premature senescence-like state (91). Recent data has shown that cellular senescence is in fact a physiological mechanism that constrains tumor development in vivo [85,86]. The signaling pathways that mediate replicative or oncogene induced senescence are not fully understood. In Mouse Embryo Fibroblasts (MEFs), p19^ARF^, which is encoded by a partially overlapping alternative reading frame at the p16^INK4A ^locus, has been implicated as a major mediator of replicative senescence. p19^ARF ^binds directly to and sequesters MDM2, thereby inhibiting the ability of MDM2 to induce degradation of the p53 tumor suppressor protein [86]. This loss of MDM2 function, in turn, results in the stabilization of p53 and activation of p53-mediated growth arrest, believed to play a major role in the irreversible growth arrest associated with the senescent phenotype. The integrity of both the p16^INK4A^/pRB and p19^ARF^/p53 pathways is essential for oncogene-induced senescence. Studies with MEFs deficient for one or more gadd45 genes, notably gadd45a, have provided evidence that gadd45 genes play important roles in both replicative and oncogene mediated senescence [61,83]. The molecular nature of stress response pathways involving Gadd45 and its interacting proteins in MEF senescence, and whether there is cross-talk with the p16^INK4A^/pRB and p19^ARF^/p53 pathways remains to be determined.

### Gadd45 as sensors of oncogenic stress

The complex role of stress sensors in monitoring oncogenic stress in tumor development is not fully understood, the best example being the multiple functions attributed to p53 in tumor development and suppression. Recent observations have implicated Gadd45 proteins as important sensors of oncogenic stress, both in vitro and in vivo.

It is known that whereas primary mouse cells require introduction of two activated oncogenes for transformation, disruption of certain key growth control genes allows single oncogene transformation [85,86]. It was shown that for MEFs obtained from gadd45a-/- mice, H-ras was sufficient for transformation [61,83]. The role gadd45b and/or gadd45g play in susceptibility of MEFs to single oncogene transformation remains to be assessed.

Evidence was obtained that Gadd45 proteins also play a role in modulation of tumor development in vivo. Gadd45a-/- and gadd45b-/- mice were observed to display increased mutation frequency, and susceptibility to ionizing radiation (IR) and chemical carcinogenesis. Also, it has been documented that NF-kB-mediated repression of gadd45a and gadd45g is essential for cancer cell survival. More recently, evidence was obtained that loss of Gadd45a accelerates ras-driven mammary tumor formation. Ras-driven tumor formation in the absence of Gadd45a resulted in both a decrease in apoptosis, linked to a decrease in JNK activation, and a decrease in senescence, correlated with a decrease in p38 kinase activation [67]. Altogether, these results provide a novel model for the tumor suppressive function of Gadd45a in the context of ras-driven breast carcinogenesis; Gadd45a elicits its function through activation of the stress induced JNK & p38 kinases, which contribute to increases in apoptosis and ras-induced senescence. How loss of gadd45a may effect breast carcinogensis driven by other oncogenes, and how loss of other gadd45 genes may contribute to breast carcinogenesis are interesting questions to be addressed in future studies.

## Conclusions & future prospects

Gadd45 genes have been implicated in stress signaling, which results in cell cycle arrest, DNA repair and cell survival, or apoptosis (Fig. 2). What deterministic factors dictate whether Gadd45 proteins function in either cell survival or apoptosis is unclear. An attractive working model to consider is that the extent of cellular/DNA damage, in a given cell type, dictates the association of different Gadd45 proteins with particular partner proteins, which determines the outcome. Future work addressing this hypothesis will undoubtedly contribute to a better understanding of how the stress sensor functions of Gadd45 proteins co-ordinate the response of cells to environmental and physiological stressors.

## Competing interests

The authors declare that they have no competing interests.

## Authors' contributions

DAL participated in the sequence alignment and drafted the manuscript. BH participated in drafting the manuscript. All authors read and approved the final manuscript.
